# Clinical Factors Associated With Trismus and the Need for Tooth Extraction in Patients With Odontogenic Abscesses: A Retrospective Clinical Study

**DOI:** 10.7759/cureus.113893

**Published:** 2026-08-03

**Authors:** Tevfik Kizilseki, Halil Ibrahim Durmus, Ümit Solmaz, Kagan Fatih Kusan, Nuran Dag, Hasan Demirkol, Adnan Oztas

**Affiliations:** 1 Oral and Maxillofacial Surgery, Faculty of Dentistry, Adıyaman University, Adıyaman, TUR; 2 Oral and Maxillofacial Surgery, Adıyaman University, Adıyaman, TUR

**Keywords:** logistic regression, maxillofacial space infection, odontogenic infection, third molar, tooth extraction, trismus

## Abstract

Objective: This retrospective study aimed to identify the clinical factors associated with the development of trismus and the need for tooth extraction in patients presenting with odontogenic maxillofacial space infections.

Materials and methods: A total of 133 patients diagnosed with odontogenic maxillofacial space infection and treated at the Department of Oral and Maxillofacial Surgery, Faculty of Dentistry, Adıyaman University, between May 2016 and May 2026 were retrospectively reviewed. Demographic characteristics, anatomical location of the infection, involved fascial spaces, causative tooth group, oral hygiene status, etiology of infection, presence of trismus, requirement for surgical drainage, and treatment modality were recorded. Factors associated with tooth extraction and trismus were analyzed using the Mann-Whitney U test, Pearson's chi-square test or Fisher's exact test, followed by multivariable logistic regression analysis.

Results: The median age of the patients was 36 years, and 52.6% were male. Dental caries was the most common cause of infection (62.1%), while the mandibular posterior region (60.2%) and the submandibular space (50.4%) were the most frequently involved anatomical sites. Trismus was observed in 45.1% of the patients, and surgical drainage was required in 78.9%. Multivariable analysis demonstrated that increasing age independently increased the likelihood of tooth extraction (OR=1.037, 95% CI: 1.008-1.067; p=0.012). Posteriorly located infections were associated with a significantly greater probability of extraction than anterior infections (OR=3.290, 95% CI: 1.116-9.705; p=0.031). Deep fascial space involvement was identified as the strongest independent predictor of trismus (OR=5.046, 95% CI: 2.091-12.175; p<0.001). In addition, infections originating from third molars showed significant associations with deep fascial space involvement, trismus, and the need for tooth extraction.

Conclusions: Advanced age and posteriorly located infections were independently associated with an increased need for tooth extraction, whereas deep fascial space involvement was the strongest predictor of trismus. Third molar-related infections demonstrated a more severe clinical presentation, characterized by higher rates of deep fascial space involvement, trismus, and tooth extraction. Early recognition of these clinical characteristics may facilitate risk stratification and support timely treatment planning in patients with odontogenic maxillofacial space infections.

## Introduction

Odontogenic infections are among the most common infections of the oral and maxillofacial region and may be associated with significant morbidity and mortality in advanced cases. The literature reports that mortality rates can reach 10-40%, especially in severe cases extending to the deep neck structures [1,2]. The oral and maxillofacial region is anatomically complex, comprising numerous interconnected fascial spaces and vital neurovascular structures. Therefore, odontogenic infections can spread rapidly, threatening airway patency and important neurovascular structures. Early diagnosis and treatment of dental caries, apical abscesses, and other odontogenic infections are essential to prevent serious complications [3].

The pattern of infection spread is mostly dependent on the anatomical localization of the originating tooth. Infections originating from the mandibular molar teeth frequently progress to the submandibular, sublingual, and pterygomandibular spaces, while infections originating from the maxillary region are more commonly seen in the buccal and canine spaces [4].

Trismus is one of the most important clinical findings of odontogenic infections and develops especially as a result of infection affecting the chewing muscles or adjacent fascial space. A decrease in maximum mouth opening can lead to impaired oral hygiene, feeding difficulties, increased pain, and difficulty in airway management. In addition, the presence of significant trismus is considered an important indicator associated with the spread of infection to deeper anatomical spaces and a more severe clinical course [5,6].

In the treatment of odontogenic fascial space infections, elimination of the source of infection, appropriate antibiotic use, and drainage when necessary are the basic treatment methods. It has been reported that timely intervention prevents the spread of infection and shortens the hospital stay [7,8].

Extraction of the infected tooth at the appropriate time is one of the fundamental steps in the treatment of odontogenic infections. Early tooth extraction has been reported to reduce the infection burden, accelerate clinical healing, and prevent the progression of infection. However, the decision to extract a tooth during the acute infection period is influenced by many clinical factors such as the degree of trismus, mouth opening, extent of infection, airway safety, and the patient's systemic status [7,9].

Most studies published to date have focused on complications, length of hospital stay, antibiotic treatment, and the need for surgical drainage in odontogenic infections. In contrast, studies evaluating the clinical factors associated with the development of trismus and the need for extraction of the infected tooth are limited. Establishing these relationships may facilitate the early identification of high-risk patients and support appropriate treatment planning [6,10].

In this study, the study population consisted of patients diagnosed with odontogenic maxillofacial space infections (Population), and no therapeutic intervention or comparison between treatment groups was predefined because of the retrospective observational design. The study evaluated the association between routinely collected clinical variables and two predefined outcomes (Outcome): the need for tooth extraction (primary outcome) and the presence of trismus (secondary outcome).

We hypothesized that deep fascial space involvement would be independently associated with the development of trismus (primary hypothesis), whereas demographic and clinical characteristics, particularly the anatomical location and patient age, would be independently associated with the need for tooth extraction (secondary hypothesis).

The aim of this retrospective study was therefore to identify the clinical factors associated with the development of trismus and the need for tooth extraction in patients with odontogenic maxillofacial space infections.

## Materials and methods

Study design and patient selection

This study was designed as a retrospective observational study. Clinical records of patients who presented to the Department of Oral and Maxillofacial Surgery, Faculty of Dentistry, Adıyaman University, with a diagnosis of odontogenic maxillofacial space infection between May 2016 and May 2026 were retrospectively reviewed. One hundred thirty-three patients with complete clinical records who met the inclusion criteria were included in the study. Patients with incomplete clinical data, infections determined not to be of odontogenic origin, and records lacking sufficient information for evaluation were excluded. Demographic and clinical data were obtained from the digital patient record systems and archive files. All data were anonymized before analysis, and patient identification information was not recorded within the scope of this study.

Sampling technique

Consecutive sampling was used because it is the recommended sampling approach for retrospective observational studies, minimizing investigator selection bias by including all eligible patients during the predefined study period. All patients diagnosed with odontogenic maxillofacial space infection who presented to the Department of Oral and Maxillofacial Surgery, Faculty of Dentistry, Adıyaman University, between May 2016 and May 2026 were screened. Patients meeting the eligibility criteria were consecutively included until all available cases had been evaluated.

Sample size

No a priori sample size calculation was performed because this was a retrospective observational study. Instead, all eligible patients treated between May 2016 and May 2026 who met the predefined inclusion criteria were included in the final analysis [11].

Inclusion criteria

Patients diagnosed with odontogenic maxillofacial space infection who were treated between May 2016 and May 2026 and had complete demographic, clinical, radiographic, and treatment records were included in the study.

Exclusion criteria

Patients with non-odontogenic infections, incomplete clinical records, insufficient radiographic documentation, recurrent infections, previous treatment at another institution before presentation, or missing key study variables were excluded.

Potential confounding variables

Potential confounding variables, including age, sex, systemic disease, oral hygiene status, infection etiology, and anatomical location, were considered during statistical modeling. Multivariable logistic regression analysis was performed to adjust for these potential confounders and to estimate their independent associations with the study outcomes.

Outcome measures

The primary outcome was the treatment modality (tooth extraction versus conservative treatment). The secondary outcome was the presence of trismus.

Data collection

For each patient, age, gender, presence of systemic disease, localization of infection in the jaw (maxilla/mandible), anatomical localization (anterior/posterior), infected fascial space, presence of trismus, need for surgical drainage, group of teeth responsible for the infection, oral hygiene status, etiology of infection, presence of cystic lesions, and applied treatment approach (tooth extraction or conservative treatment) were recorded. Trismus was defined as a clinically documented limitation of mouth opening recorded during the initial examination by the attending oral and maxillofacial surgeon. Patients were classified as having trismus only when this finding was explicitly documented in the medical record. In the analyses, infection sites were also grouped according to anatomical spread as deep space infections (submandibular, sublingual, and submental spaces) and superficial space infections (buccal, buccopalatal, palatal, and canine fossa). The primary endpoint of the study was the treatment approach (tooth extraction or conservative treatment), and the secondary endpoint was the presence of trismus.

Statistical analysis

Data from the 133 included patients were summarized using descriptive statistics, including frequencies, percentages, medians, and interquartile ranges (IQRs). The distribution of continuous variables was assessed using the Shapiro-Wilk test. As continuous variables were not normally distributed, comparisons between groups were performed using the Mann-Whitney U test. Associations between categorical variables were evaluated using Pearson's chi-square test, while Fisher's exact test was applied for 2×2 contingency tables and when more than 20% of the expected cell counts were less than 5.

Binary logistic regression analyses were performed to identify independent factors associated with the need for tooth extraction (vs. conservative treatment) and the presence of trismus. Candidate variables were initially screened using univariable analyses. Variables with a p-value <0.20 and those considered clinically relevant were subsequently entered into multivariable logistic regression models after assessment of multicollinearity using the variance inflation factor (VIF).

Model performance was assessed using the Hosmer-Lemeshow goodness-of-fit test and the area under the receiver operating characteristic (ROC) curve (AUC). The results are presented as odds ratios (ORs) with corresponding 95% confidence intervals (95% CIs).

Because a small number of observations (1-2 cases) contained missing data for systemic disease, oral hygiene status, infection etiology, cystic lesion status, and treatment modality, analyses were performed using available-case analysis (listwise/pairwise deletion), and the sample size for each analysis is reported in the corresponding tables. A two-sided p-value <0.05 was considered statistically significant. All statistical analyses were performed using Python 3.14.6 (Python Software Foundation, Fredericksburg, VA, USA) with SciPy, statsmodels, and scikit-learn libraries.

## Results

A total of 133 patients with odontogenic maxillofacial space infections who met the inclusion criteria were included in the analysis. The demographic and clinical characteristics of the study population are summarized in Table 1.

**Table 1:** General demographic and clinical characteristics of the study population (n=133). Data are presented as n (%) or median (IQR). Values marked with an asterisk (*) contain one missing observation; percentages were calculated using the available data. One patient with missing treatment information was excluded from comparisons between the tooth extraction and conservative treatment groups (n=132). IQR: interquartile range (25th-75th percentile).

Variable	Category	Value
Age (years)	Median (IQR)	36 (27–45)
	Range	11–88
Sex	Male	70 (52.6)
	Female	63 (47.4)
Systemic disease*	Present	9 (6.8)
	Absent	123 (93.2)
Anatomical location	Mandibular posterior	80 (60.2)
	Maxillary posterior	28 (21.1)
	Maxillary anterior	17 (12.8)
	Mandibular anterior	8 (6.0)
Involved fascial space	Submandibular	67 (50.4)
	Buccal	31 (23.3)
	Sublingual	13 (9.8)
	Submental	8 (6.0)
	Canine fossa	8 (6.0)
	Buccopalatal	3 (2.3)
	Palatal	3 (2.3)
Trismus	Present	60 (45.1)
	Absent	73 (54.9)
Need for surgical drainage	Yes	105 (78.9)
	No	28 (21.1)
Causative tooth group	Molar	67 (50.4)
	Premolar	26 (19.5)
	Anterior (incisor/canine)	25 (18.8)
	Third molar (wisdom tooth)	15 (11.3)
Oral hygiene status*	Poor	83 (62.9)
	Fair	45 (34.1)
	Good	4 (3.0)
Etiology of infection*	Dental caries	82 (62.1)
	Failed root canal treatment	21 (15.9)
	Periodontal disease	16 (12.1)
	Pericoronitis	8 (6.1)
	Dental caries + periodontal disease	5 (3.8)
Cystic lesion*	Present	92 (69.7)
	Absent	40 (30.3)
Treatment modality*	Tooth extraction	74 (56.1)
	Conservative treatment	58 (43.9)

The median age of the study population was 36 years (IQR: 27-45; range: 11-88). Of the 133 patients, 70 (52.6%) were male and 63 (47.4%) were female. The source of infection in the majority of cases was dental caries (82 patients, 62.1%), with the most frequently affected area being the posterior mandible (80 patients, 60.2%) and the most common site of infection being the submandibular space (67 patients, 50.4%). Trismus was present in 60 patients (45.1%), and oral hygiene was assessed as poor in 83 patients (62.9%) (valid n=132). The causative tooth was in the molar group in 67 patients (50.4%); 15 patients (11.3%) had infections originating from impacted or partially impacted third molars. Of the 132 patients with available data, 74 (56.1%) underwent tooth extraction, and 58 (43.9%) received conservative treatment (root canal treatment, root canal restoration, or apical resection).

The associations between treatment modality (tooth extraction vs. conservative treatment) and demographic and clinical variables are presented in Table 2. 

**Table 2:** Comparison of clinical characteristics between extraction and conservative treatment groups. Data are presented as n (%) or median (IQR). Comparisons were performed using the Mann-Whitney U test for continuous variables and Pearson's chi-square test or Fisher's exact test for categorical variables, as appropriate. Because the expected cell count was less than 5 in some cells for the involved fascial space and oral hygiene status variables (minimum expected counts: 1.32 and 1.76, respectively), the corresponding chi-square test results should be interpreted with caution. IQR: interquartile range; U: Mann-Whitney U test; χ²: Pearson's chi-square test; df: degrees of freedom. ^*^Statistical significance was defined as p<0.05. ^†^Fisher's exact test was used.

Variable	Category	Tooth extraction (n=74)	Conservative treatment (n=58)	Test statistic	p-value
Age (years)	Median (IQR)	37 (28.25–47.5)	32 (21.25–45)	U=2495.5	0.109
Sex	Male	39 (52.7)	30 (51.7)	Fisher's exact	1.000^†^
	Female	35 (47.3)	28 (48.3)		
Systemic disease	Present	4 (5.4)	5 (8.6)	Fisher's exact	0.505^†^
	Absent	70 (94.6)	53 (91.4)		
Anatomical location	Mandibular anterior	3 (4.1)	5 (8.6)	χ²=10.975 (df=3)	0.012*
	Mandibular posterior	53 (71.6)	26 (44.8)		
	Maxillary anterior	5 (6.8)	12 (20.7)		
	Maxillary posterior	13 (17.6)	15 (25.9)		
Involved fascial space	Submandibular	45 (60.8)	21 (36.2)	χ²=10.380 (df=6)	0.110
	Buccal	12 (16.2)	19 (32.8)		
	Sublingual	8 (10.8)	5 (8.6)		
	Submental	3 (4.1)	5 (8.6)		
	Canine fossa	4 (5.4)	4 (6.9)		
	Buccopalatal	1 (1.4)	2 (3.4)		
	Palatal	1 (1.4)	2 (3.4)		
Trismus	Present	39 (52.7)	20 (34.5)	Fisher's exact	0.052^†^
	Absent	35 (47.3)	38 (65.5)		
Need for surgical drainage	Yes	57 (77.0)	47 (81.0)	Fisher's exact	0.670^†^
	No	17 (23.0)	11 (19.0)		
Oral hygiene status	Poor	51 (68.9)	32 (55.2)	χ²=4.025 (df=2)	0.134
	Fair	20 (27.0)	25 (43.1)		
	Good	3 (4.1)	1 (1.7)		

The median age of the tooth extraction group tended to be higher compared to the conservative treatment group, but the difference did not reach statistical significance (Mann-Whitney U=2495.5, p=0.109). A statistically significant relationship was found between the anatomical region of the infection and the treatment approach (χ²=10.975, df=3, p=0.012); the tooth extraction rate was significantly higher in posterior mandibular involvement (71.6%) compared to the conservative treatment, while the conservative treatment rate was higher in anterior maxillary involvement (20.7%). The relationship between the presence of trismus and treatment preference showed borderline significance (Fisher's exact test, p=0.052); the tooth extraction rate was higher in patients with trismus (52.7%) compared to those without trismus (34.5%). No statistically significant differences were found between the groups in terms of gender, presence of systemic disease, need for drainage, oral hygiene status, cause of infection, location of infection, and presence of cystic lesions (p>0.05 for all).

Variables independently associated with the need for tooth extraction were identified using multivariable logistic regression analysis, and the final model is presented in Table 3.

**Table 3:** Final multivariate logistic regression model after variable selection. Results are presented as B, OR, 95% CI, and p-value. The dependent variable was tooth extraction (1) versus conservative treatment (0). The reference categories were mandible (jaw location), anterior location, absence of trismus, and non-caries-related infections (infection etiology). Model fit was assessed using the likelihood ratio chi-square test (χ²=22.36, df=5, p<0.001), with a McFadden's pseudo-R² of 0.124. No evidence of multicollinearity was observed, as all VIF values were <1.25. B: regression coefficient; OR: odds ratio; CI: confidence interval; VIF: variance inflation factor. ^*^Statistically significant (p<0.05).

Variable	B	OR	95% CI	p-value
Intercept	-1.544	0.214	0.043–1.058	0.059
Age (years)	0.036	1.037	1.008–1.067	0.012*
Jaw (maxilla vs. mandible)	-0.826	0.438	0.183–1.046	0.063
Location (posterior vs. anterior)	1.191	3.290	1.116–9.705	0.031*
Trismus (present vs. absent)	0.583	1.791	0.773–4.147	0.174
Etiology of infection (dental caries vs. others)	-0.769	0.464	0.207–1.039	0.062

In the final multivariable logistic regression model, age was identified as a significant and independent predictor of the need for tooth extraction (OR=1.037, 95% CI: 1.008-1.067, p=0.012). Anatomical position also emerged as an independent and significant predictor (OR=3.290, 95% CI: 1.116-9.705, p=0.031); a posterior location increased the probability of extraction approximately 3.3 times. Jaw location (OR=0.438, p=0.063) and cause of infection (caries vs. other; OR=0.464, p=0.062) remained at borderline significance, while maxillary location and caries-related cases were associated with a tendency towards conservative treatment. The presence of trismus was not found to be a statistically significant independent predictor in the model (OR=1.791, p=0.174). The model's discriminative power was acceptable (AUC=0.745), and the Hosmer-Lemeshow test confirmed that the model fitted the observed data well (p=0.861); no multicollinearity was found between the variables (VIF<1.25).

Multivariable logistic regression analysis was also performed to identify independent predictors of trismus. The results of the final model are summarized in Table 4.

**Table 4:** Final multivariate logistic regression model for trismus. Results are presented as B, OR, 95% CI, and p-value. The dependent variable was trismus (1) versus no trismus (0). The reference category was superficial fascial space involvement. Model fit was assessed using the likelihood ratio chi-square test (χ²=26.83, df=3, p<0.001), with a McFadden's pseudo-R² of 0.148. No evidence of multicollinearity was observed, as all VIF values were <1.19. B: regression coefficient; OR: odds ratio; CI: confidence interval; VIF: variance inflation factor. ^*^Statistically significant (p<0.05).

Variable	B	OR	95% CI	p-value
Intercept	0.584	1.793	0.405-7.938	0.442
Age (years)	-0.022	0.979	0.951-1.007	0.134
Involved fascial space (deep vs. superficial)	1.619	5.046	2.091-12.175	<0.001*
Oral hygiene status (ordinal; Good → Poor, per one-category increase)	-0.710	0.492	0.232-1.040	0.063

In the multivariate model, the depth of the infection space was confirmed as the strongest and most consistent independent predictor of the presence of trismus (OR=5.05, 95% CI: 2.09-12.18, p<0.001); the risk of trismus was approximately five times higher in patients with deep space (submandibular/sublingual/submental) involvement compared to those with superficial space involvement. Oral hygiene was found to be borderline significant in the multivariate analysis (OR=0.49; p=0.063). This finding may be related to possible confounding effects depending on the etiology of the infection and the causative tooth group.

Relationship between the causative tooth group and trismus, deep fascial space involvement, and treatment approach

The causative tooth was divided into four clinical groups according to its position number in the FDI tooth numbering system: anterior (incisor/canine, positions 1-3), premolar (positions 4-5), molar (positions 6-7 and primary molars), and third molar/wisdom tooth (position 8). The relationship between this grouping and the presence of trismus, the depth of the infection cavity (deep/superficial), and the indication for treatment (extraction/conservative treatment) was evaluated using Pearson chi-square test and Cramér's V effect size.

The relationships between the causative tooth group and trismus, deep fascial space involvement, and treatment modality were further evaluated. The distribution of these variables according to tooth group is presented in Table 5.

**Table 5:** Distribution of trismus, deep fascial space involvement, and treatment indication according to the causative tooth group. Pearson's chi-square test was used. Effect size was assessed using Cramér's V. For all analyses, the minimum expected cell count exceeded 4.7, indicating that the assumptions of the chi-square test were satisfied. Statistical significance was defined as p<0.05. Trismus: χ²=42.751, df=3, p<0.001, Cramér's V=0.567 (strong association), n=133. Deep fascial space involvement: χ²=37.209, df=3, p<0.001, Cramér's V=0.529 (strong association), n=133. Tooth extraction: χ²=18.245, df=3, p<0.001, Cramér's V=0.372 (strong association), n=132 (one patient with unknown treatment modality was excluded from the third molar group). ^*^The percentage for tooth extraction was calculated based on the 14 patients with available treatment data (14/14).

Tooth group	n	Trismus, n (%)	Deep fascial space involvement, n (%)	Tooth extraction, n (%)
Anterior (incisor/canine)	25	3 (12.0)	8 (32.0)	8 (32.0)
Premolar	26	3 (11.5)	10 (38.5)	12 (46.2)
Molar	67	40 (59.7)	55 (82.1)	40 (59.7)
Third molar (wisdom tooth)	15	14 (93.3)	15 (100.0)	14 (100.0)^*^

The causative tooth group was found to be statistically significant and strongly associated with all three clinical outcomes examined. All cases originating from the third molar (wisdom tooth) showed deep fascial space involvement (100%), and extraction was performed in all cases with valid data (14/14). Furthermore, the incidence of trismus in this group (93.3%) was significantly higher than that in all other groups; this finding was associated with a more severe clinical course and a greater need for tooth extraction in pericoronitis and secondary deep space infections due to impacted/partially impacted third molars. The molar group ranked second with relatively high rates of trismus (59.7%) and deep fascial space involvement (82.1%), whereas cases originating from anterior and premolars showed significantly lower rates of both trismus and deep fascial space involvement (11-12% and 32-39%, respectively) and a higher rate of conservative treatment. These findings indicate that posterior infections, particularly those originating from the third molar, are associated with deep fascial space involvement, trismus, and the need for tooth extraction.

## Discussion

In this retrospective study, clinical factors associated with the need for tooth extraction and the development of trismus were evaluated in 133 patients treated for maxillofacial space infections of odontogenic origin. Our findings show that advanced age and posterior location are associated with the need for tooth extraction, and deep fascial space involvement is the strongest independent predictor of trismus development. Furthermore, deep fascial space involvement, trismus, and tooth extraction rates were found to be higher in third molar-related infections compared to other tooth groups.

The demographic and clinical profile of our study population (median age 36, male/female ratio approximately 1.1/1, submandibular space being the most frequently affected space) largely coincides with large-scale series in the literature. Gadicherla et al. reported a mean age of 38.6 in their series of 128 patients and identified the submandibular space as the most frequently affected area (59 patients, 46.1%) [12]. A similar distribution was observed in Zhang et al.'s Chinese series of 212 cases [13]. In a series reported from Türkiye (Ekici, two-year retrospective evaluation), the epidemiological profile of odontogenic fascial space infections was similarly described [14]. These findings show that the demographic and clinical characteristics of our study population are consistent with previous studies.

The independent increase in the probability of extraction due to age (Table 3; OR=1.037, p=0.012) can be explained by periodontal support loss, decreased pulpal/periapical healing capacity, and lower tolerance to conservative endodontic treatment in older patients. This finding is consistent with the general maxillofacial infection literature, where advanced age stands out as a negative prognostic indicator; indeed, Gadicherla et al. reported advanced age as an independent predictor of prolonged hospital stay [12], and in the meta-analysis of 12 studies by Wang et al., age was also found to be a significant risk factor for the development of complications (OR=1.59) [14]. In our study, the effect of age was shown not through hospital stay or complications, but directly through the treatment decision (extraction). In this respect, it provides a complementary finding to the literature showing the role of the effect of age in the clinical decision-making process. The strong association we found between trismus and deep fascial space involvement (Table 4; OR=5.05, p<0.001) is consistent with the knowledge that trismus is pathophysiologically associated with inflammatory involvement of the deep spaces (submandibular, sublingual, submental) adjacent to the masticatory muscles (especially the pterygoid and masseter muscles). Similarly, in Zhang et al.'s large series of 408 cases, trismus was found to be one of the strongest independent predictors of the severity of oral and maxillofacial space infections (OR=4.225), and remarkably, the "odontogenic origin" variable lost its significance in the multivariate model; the authors explained this by the fact that trismus and dysphagia are mediator clinical variables [15]. Our findings support the idea that deep fascial space involvement is an important clinical indicator in the development of trismus.

The fact that all cases originating from the third molar showed deep fascial space involvement and resulted in extraction (Table 5) is consistent with classical anatomical studies showing that infection tends to spread directly to the submandibular and sublingual spaces due to the anatomical location of the mandibular 3rd molar roots below the mylohyoid muscle and medial to the lingual cortex. Thomas Indresano et al. were the first to demonstrate that third molars are a significant cause of deep space infections [16]. Ariji et al. confirmed the passage of pericoronitis into the submandibular space by visualizing these spread pathways with computed tomography [17]. In our study, the strong association of third molar-derived infections with deep fascial space involvement, trismus, and the need for tooth extraction clinically supports this anatomical information.

Our study has some important limitations. Firstly, the single-center retrospective design carries an inherent risk of selection bias and unmeasured confounding. In addition, because the study relied on retrospective medical records, information and measurement bias may have occurred, particularly in the documentation of trismus and the clinical assessment of oral hygiene. Furthermore, pre-presentation antibiotic use, differences in imaging selection, and inter-surgeon variability in clinical decision-making may have influenced the recorded clinical findings and treatment decisions. Secondly, the sample size (n=133) and especially the small number of patients in the third molar group may have resulted in wide confidence intervals for some estimates. Thirdly, our definition of "deep space" was limited to the submandibular, sublingual, and submental areas and did not include other deep spaces adjacent to the masticatory muscles, such as the pterygomandibular, masseteric, and temporal, which are associated with trismus in the literature [15,18]. It should be noted that this classification was made according to the existing categories in the dataset, and a more detailed anatomical subclassification could be addressed in future studies. Finally, these findings need to be validated in different centers with larger samples [19].

## Conclusions

In conclusion, advanced age, posterior location, and especially third molar-originating infections were found to be associated with the need for tooth extraction, while deep fascial space involvement was identified as the strongest independent predictor of trismus development. These findings may contribute to early risk assessment and treatment planning in patients with odontogenic infections. Multicenter prospective studies with larger sample size are needed to confirm these findings.

The findings of this retrospective study demonstrate that the most important factors influencing treatment decisions in odontogenic maxillofacial space infections are advanced age, posterior location of infections, and especially infections originating from the third molar. Furthermore, deep fascial space involvement was identified as the strongest independent predictor of trismus development. Early recognition of these clinical features can contribute to identifying high-risk patients, planning appropriate surgical approaches in a timely manner, and optimizing the treatment process. However, due to the retrospective and single-center design of the study, the findings need to be validated in different populations. Supporting these findings with larger multicenter prospective studies will improve their reliability and generalizability.
